# Effects of Diethylstilbestrol on Zebrafish Gonad Development and Endocrine Disruption Mechanism

**DOI:** 10.3390/biom11070941

**Published:** 2021-06-25

**Authors:** Xuan Liu, Xianyi Xie, Hongling Liu

**Affiliations:** State Key Laboratory of Pollution Control and Resource Reuse, School of the Environment, Nanjing University, Nanjing 210023, China; mg20250024@smail.nju.edu.cn (X.L.); mf1725069@smail.nju.edu.cn (X.X.)

**Keywords:** environmental estrogen, diethylstilbestrol (DES), zebrafish gonad development, endocrine disruption-chemicals (EDCs)

## Abstract

Environmental estrogen is a substance that functions as an endocrine hormone in organisms and can cause endocrine system disruption. A typical environmental estrogen, diethylstilbestrol (DES), can affect normal sexual function and organism development. However, even though the effects of different exposure stages of DES on the endocrine system and gonadal development of zebrafish juveniles are unknown, sex determination is strongly influenced by endocrine-disrupting chemicals (EDCs). From 10–90 days post fertilization (dpf), juvenile zebrafish were exposed to DES (100 and 1000 ng/L) in three different stages (initial development stage (IDS), 10–25 dpf; gonadal differentiation stage (GDS), 25–45 dpf and gonadal maturity stage (GMS), 45–60 dpf). Compared with that of IDS and GMS, the growth indicators (body length, body weight, and others) decreased significantly at GDS, and the proportion of zebrafish females exposed to 100 ng/L DES was significantly higher (by 59.65%) than that of the control; in addition, the zebrafish were biased towards female differentiation. The GDS is a critical period for sex differentiation. Our results show that exposure to environmental estrogen during the critical gonadal differentiation period not only affects the development of zebrafish, but also affects the population development.

## 1. Introduction

In recent decades, plenty of evidence from laboratory animals, in vitro studies, and epidemiology showed that many synthetic environmental estrogens that are released into the environment have the capacity to imitate the actions of the natural hormone 17β-estradiol (E2), which then causes adverse outcomes for humans and animals [1,2,3]. Diethylstilbestrol (DES), a nonsteroidal estrogen, was the first synthetic estrogen used in clinical treatment to prevent miscarriages. In 1971, the U.S. Food and Drug Administration (FDA) issued the ‘Drug Bulletin’, advising doctors to stop prescribing DES to pregnant women because it was related to a rare vaginal cancer in female offspring [4]. However, DES is still used in Asia and other regions as a growth promoter for livestock [5,6]. It is also used in aquaculture for promoting growth and for producing monosex (female) populations of fish [7]. According to reports, the detectable concentration of DES in surface water ranges from slightly less than 1 to about 20 ng/L in different locations [8,9,10]. In China, the concentration of DES in some wastewaters, as well as in wastewater from wastewater treatment plants, reached 421 and 268 ng/L, respectively [11].

Recently, a toxicology study found some adverse developmental effects caused by DES. Some studies [12,13,14,15] found that DES had a damaging impact on male Sprague–Dawley rattos’ reproductive systems through decreasing the numbers of spermatogonia, Sertoli cells, and Leydig cells, which affect steroidogenesis and spermatogenesis. Estrogenic endocrine-disrupting chemical (EDC) exposure, including to E2, DES, mestranol, and 4-n-nonyphenol, disrupts testis differentiation and causes xenopus laevis feminization to different degrees [16]. Air-breathing catfish exposed to 17α-ethynylestradiol (EE2) and DES presented with a developmental delay, as well as premature morphological and oocyte development during the early stages of growth and gonadal differentiation [17]. Wang et al. [18] observed that the oogenesis of yellow catfish juveniles were affected by DES by disrupting gonad development and the hypothalamic-pituitary-gonadal (HPG) axis. Pan et al. [19] found that mature male zebrafish exposed to DES and flutamide for 30 days had damaged spermatogenesis. In the present study, we attempted to determine when the sensitive development and endocrine-disrupting effect stage of environmental estrogen DES occurred for aquatic organisms. A previous study [18] found that 10 days of DES exposure could disrupt the HPG axis, promote apoptosis and meiosis, and impair the oogenesis of female yellow catfish juveniles. However, despite the available research [5,18,19] based on experiments involving DES exposure in mature fish, there is a lack of evidence about the specific stages of DES exposure. The zebrafish is an ideal model organism and is used in a large number of toxicological studies [20], as its gonadal differentiation style is dioecious and its sex is not determined at birth, as zebrafish gradually differentiate to have male or female gonads during their individual growth and development. Environmental sex determination (ESD) plays a crucial role in zebrafish sex differentiation [21]. According to previous research, we can divide zebrafish gonad development into three phases: (1) the initial development stage (IDS), which is about 10–25 days post fertilization (dpf), where the zebrafish gonad develops into an ovarian gonad and primordial germ cells begin to undergo mitosis [22]; (2) the gonadal differentiation stage (GDS), which is at 25–45 dpf and is an important turning point in gonadal differentiation [23], and (3) the gonadal maturity stage (GMS), which occurs at 45–60 dpf, after which complete gonad differentiation can be observed at 60 dpf and the gonad will be completely sex mature [24]. Therefore, to better understand the gonad development toxicity of DES in zebrafish, three phases, according to their development characteristics, were chosen for exposure applying two concentrations (100 and 1000 ng/L) until complete maturity was maintained at 90 dpf.

## 2. Materials and Methods 

### 2.1. Materials

#### 2.1.1. Chemicals

DES (CAS number 56-53-1) was purchased from Shanghai J&K Scientific (Shanghai, China) and the purity was above 95%. Acetone (CAS number 67-64-1) was purchased from Shanghai Generay Biotech (Shanghai, China) and the purity was above 99%.

#### 2.1.2. Zebrafish Maintenance

Zebrafish (Danio rerio) embryos were obtained from the same batch of 4-month-old wild AB-type adult zebrafish, raised in a laboratory zebrafish breeding system, through natural mating at a 1:2 ratio of female to male. The embryos were maintained at 28 ± 1 °C in 60 mg/L of sea salt water (pH = 6.5–8.5, dissolved oxygen ≥ 60% saturation) with a 14/10 h light/dark cycle. The fish were fed thrice daily with fairy shrimp (Tianjin Red Sun Aquaculture Co. Ltd. Tianjin, China). The amount of food did not exceed 2% of the body weight.

### 2.2. Methods

#### 2.2.1. Eighty-Day Exposure Experiments

**Figure A1:**
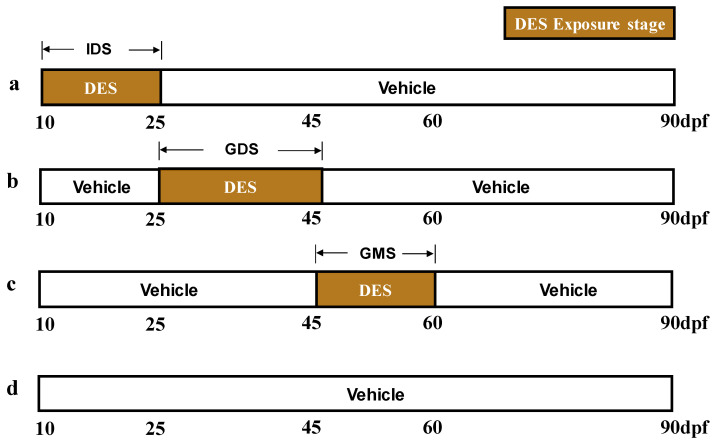
Different stages of DES exposure: (**a**) zebrafish exposed to DES from 10 to 25 dpf, which is initial development stage (IDS); (**b**) zebrafish exposed to DES from 25 to 45 dpf, which is gonadal differentiation stage (GDS); (**c**) zebrafish exposed to DES from 45 to 60 dpf, which is gonadal maturity stage (GMS); (**d**) zebrafish exposed to 0.01% acetone. We only exposed zebrafish at IDS, GDS, and GMS, individually, and maintained them in freshwater for 90 dpf after exposure phase.

We investigated the concentration of DES on the surface and in wastewater [8,9,10,11], and then we did a pretest to find an appropriate concentration that would affect the development of zebrafish, but with no serious outcomes. Consequently, two doses (100 and 1000 ng/L) of DES were chosen and used for the exposure in this study. To assess the effects of DES exposure in different gonad development stages, we conducted experiments in three exposure periods (as illustrated in Figure A1 in Appendix A) according to the gonad development process of zebrafish [24]. Specifically, zebrafish larvae were only exposed to DES in IDS, GDS, and GMS separately, and were maintained in 0.01% acetone for 90 dpf, after the exposure phase (as illustrated in Figure A1). The exposure started on 10 dpf after adult fish mating. The treatment and control groups were prepared in triplicate. Each replication had 50 zebrafish larvae, which were randomly selected and placed in 2 L glass beakers with a DES solution (0, 100, and 1000 ng/L, dissolved in 0.01% acetone) from 10 to 25 dpf, and then moved into a 5 L glass beaker to ensure that they had enough living space after 25 dpf (age < 30 dpf, space < 30 fish/L; age > 30 dpf, space < 5 fish/L). During the exposure, the water in each glass beaker was replaced every two days with freshly exposed water containing the corresponding drug concentrations. To avoid the evaporation of the solution, each glass beaker was covered with a breathable membrane during the experiment. We measured some of the morphological parameters (body length (L), body weight (W), and sex) of the zebrafish exposed at 90 dpf and also the body length of the larvae at 25, 45, and 60 dpf in the experimental period. The body length and body weight were measured according to the Organization for Economic Cooperation and Development (OECD) 236 guidelines and the Fish Early-Life Stage Toxicology Test (OECD 210, 2013). In the below equation, K indicates plumpness, which was calculated as described in Formula (1), which was used to estimate the true growth rates.

K = W/L^3^ × 100 (1)

In addition, we also recorded the sexual distribution in different groups to observe the effect of DES on the sex ratio. At 90 dpf, the zebrafish were placed in ice water to euthanize them and confirm the sex of each zebrafish by the dissection of the gonads, thereby enabling us to determine the sex ratio statistics. The gonads were weighed (W_gonad_), and the gonadosomatic index (GSI) was calculated as described in Formula (2).

GSI = W_gonad_/W × 100 (2)

#### 2.2.2. Observation of Gonadal Tissue

To study the effects of exposure to DES over three different periods on zebrafish gonad development and to understand the maturity of zebrafish gonad development at 90 dpf, we observed a gonadal tissue section and judged the sex of the fish according to the OECD No.123 Guidance Document on the Diagnosis of Endocrine-Related Histopathology in Fish Gonads (2010). We also counted the proportion of germ cells in the gonads of male and female zebrafish at various developmental stages.

The gonadal tissues sampled from 4 fish for every replicate at 90 dpf were randomly selected and dissected. We rinsed the gonads with normal saline, immersed them in a 10% formaldehyde solution and placed them in a cool environment at room temperature for more than 48 h, and then conducted tissue slice observations on the gonads by paraffin sectioning and hematoxylin-eosin (HE) staining. The female gonadal section was divided into several areas 1000 × 1000 μm in size; we took 2–3 areas of each slice from repeat experiments of each exposure and control groups to count the number of egg cells. When there was an atresia follicle, a degenerated oocyte with a separated cell content and vitelline membrane, the statistics were not calculated but a record was made. Egg cells in females mainly include perinuclear oocytes (PO), cortical granular oocytes (CO), early vitellogenesis oocytes (EV), and late vitellogenesis oocytes (LV), and sperm cells in males include spermatogonia (sg), spermatocytes (sc), spermatids (st), and sperm (sp). As male germ cells are too small to be counted, we counted the area of the sperm cells at each stage. Besides the male gonad slices, which were divided into several 200 × 200 μm areas, the sizes of these slices were the same as those mentioned before.

#### 2.2.3. Analysis of Genes and Proteins

The gene expression analyses were conducted using the qRT-PCR method. A qRT-PCR assay of the genes on the HPG axis and the genes related to ER (the estrogen receptor) and AR (the androgen receptor) was conducted to assess the triggers of reproductive toxicity in zebrafish. Three fish from each replicate were selected to extract RNA from the control group (at 25, 45, 60, and 90 dpf), and the IDS (at 25 dpf, 45 dpf (only 1000 ng/L)), GDS (at 45 dpf and 60 dpf (only 1000 ng/L)), and GMS (at 60 dpf and 90 dpf (only 1000 ng/L)) exposure groups, and the samples were then stored in RNAlater (Qiagen, Hilden, Germany) at −80 °C in a refrigerator. Three zebrafish larvae were pooled and homogenized as one biological sample. The total RNA was extracted using an RNeasy Mini kit (Qiagen, Hilden, Germany), and the cDNA was transcribed with an Omniscript RT kit (Qiagen, Germany) according to the manufacturer’s instructions. A SYBR real-time quantitative PCR kit from Toyobo (Shanghai, China) Biotechnology (Shanghai, China) was used to amplify the gene expressions related to the HPG axis, ER, and AR. The sequences of the selected genes and primers were characterized previously [25] and are listed in Table 1. The housekeeping gene beta-actin was used as an internal control for the mRNA expression, and the gene expression was calculated with the 2 ^−ΔΔCt^ method [26]. At 25 and 45 dpf, the measured genes included those related to growth, the zebrafish HPG axis, the sex hormone receptor, sex differentiation, and gonad development. At 60 and 90 dpf, because the gonadal differentiation process ended, the tested genes included those related to the HPG axis and the zebrafish sex hormone receptor.

#### 2.2.4. Statistical Analysis

The toxicology data in this study were statistically analyzed using Graph Pad Prism5 (GraphPad, San Diego, CA, USA). There was no significant difference between the water control and the solvent control for all investigated endpoints, and thus, the solvent control was used as the control in the following analysis. A one-way analysis of variance (one-way ANOVA) was applied to analyze the significance of differences between the exposed group and the control group at each key timepoint. Values are shown as the mean ± standard deviation (SD). Significant differences were found when *p* < 0.05.

## 3. Results

### 3.1. Morphology Parameters

#### 3.1.1. 25,45 and 60 dpf Body Length

**Figure 1:**
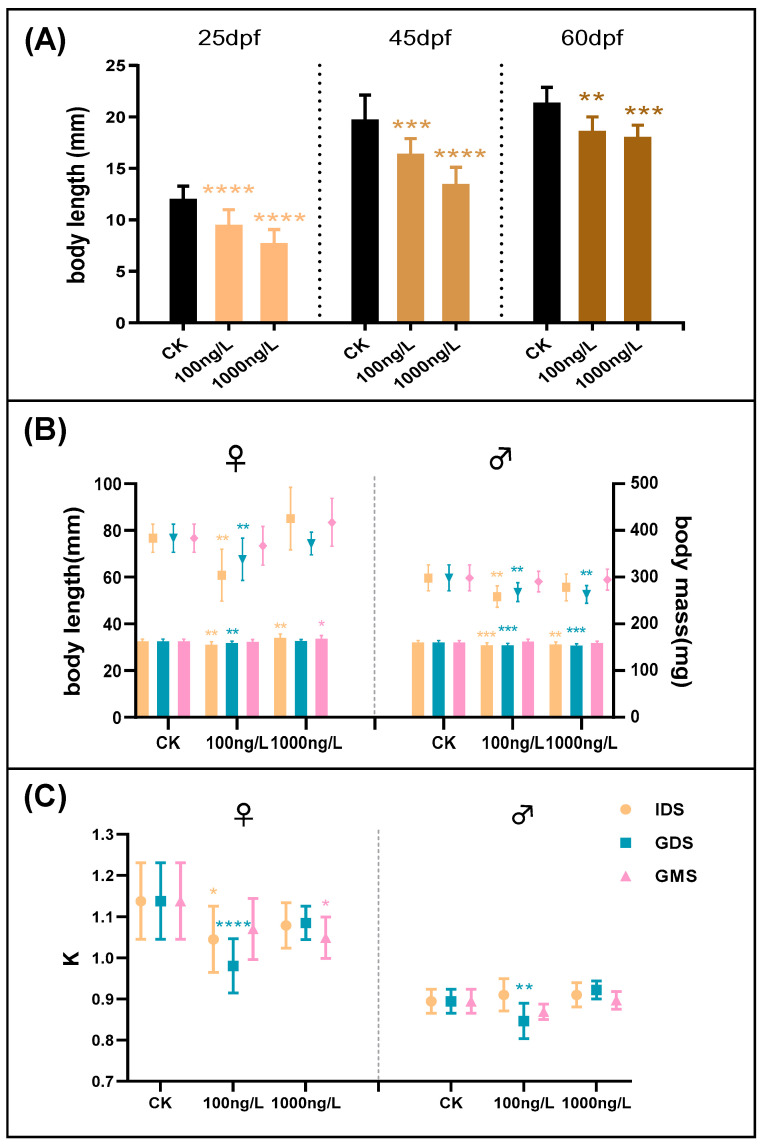
(**A**) Body length measured after diethylstilbestrol (DES) exposure at 25 dpf, 45 dpf, and 60 dpf, respectively. (**B**) Body length and body weight of zebrafish maintained after exposure, measured at 90 dpf. Different shapes of dots are body weight, and histogram is the body length (yellow is initial development stage (IDS), blue is gonadal differentiation stage (GDS), and pink is gonadal maturity stage (GMS)). (**C**) The plumpness (K) of zebrafish maintained after exposure, measured at 90 dpf. Values are shown as the mean ± SD (*n* = 3 replicates per treatment, *n* = 11 fish per replicate at 25, 45, and 60 dpf, and *n* = 15 fish per replicate at 90 dpf). Asterisks denote significant differences between control and treatments (* *p* < 0.05, ** *p* < 0.01, *** *p* < 0.005, and **** *p* < 0.0001, according to Dunnett’s multiple comparisons test). CK-control group.

The body length after every exposure stage was significantly reduced compared with that of the control, which demonstrated that DES exposure in the development stage had a significant inhibitory effect on zebrafish body development, as shown in Figure 1. At 25 dpf, the body length of the fish who were in the IDS when they were exposed was significantly lower than in that of the control groups (0 ng/L; lower by 20.91% (*p* < 0.0001) and 35.29% (*p* < 0.0001)) for the 100 ng/L and 1000 ng/L treatments, respectively. At 45 dpf, the body length of fish subjected to GDS exposure was significantly lower (by 16.74% (*p* < 0.001) and 31.57% (*p* < 0.0001)) in the 100 ng/L and 1000 ng/L treatment groups than in that of the control groups, respectively. At 60 dpf, the body length of fish that underwent GMS exposure was significantly lower (by 12.79% (*p* < 0.01) and 15.40% (*p* < 0.001)) for the 100 ng/L and 1000 ng/L treatments, respectively.

##### 3.1.2. Body Length, Body Weight, and K at 90 dpf

**Figure 2:**
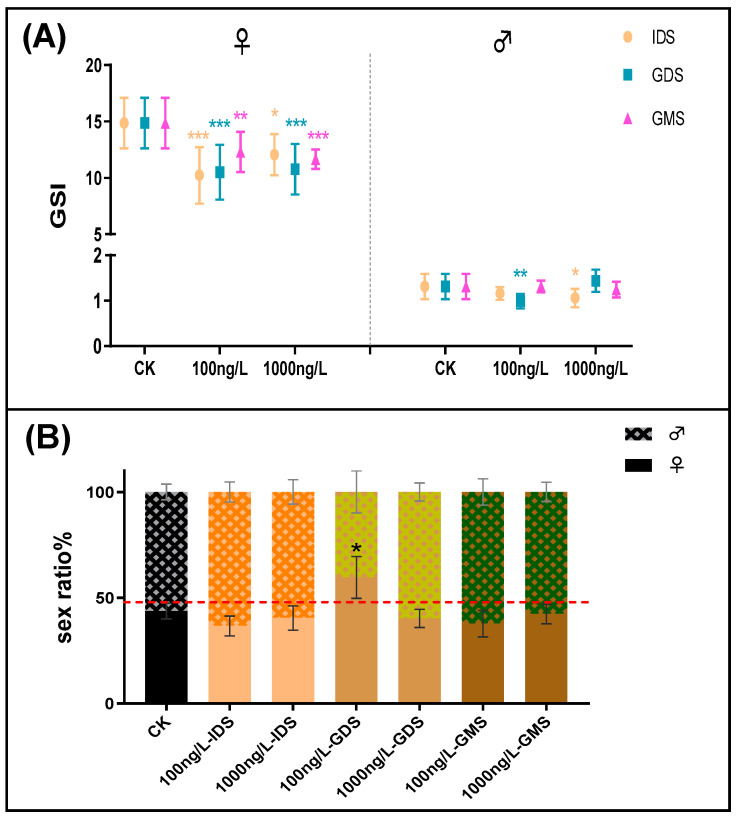
(**A**) Gonadosomatic index (GSI) of zebrafish at 90 dpf for every group. (*n* = 15 fish per replicate at 90 dpf). (**B**) Sex ratio of zebrafish at 90 dpf for every group. (*n* = 6 fish per replicate at 90 dpf). Values are shown as mean ± SD (*n* = 3 replicates per treatment). Asterisks denote significant differences between control and treatments (* *p* < 0.05, ** *p* < 0.01, and *** *p* < 0.005, according to Dunnett’s multiple comparisons test). CK-control group.

We found that exposure to DES in different development stages affected multiple aspects of zebrafish development, including body length, body weight, K, and sex (as illustrated in Figure 1C and Figure 2A). The zebrafish that were exposed to DES in the IDS showed a significant decrease in body length at 25 dpf (as illustrated in Figure 1B). At 90 dpf, the body length and body weight were still significantly decreased when the zebrafish were kept in a freshwater environment for 65 days after exposure in the IDS (100 ng/L) and were sexually mature. However, the body length of females exposed during the IDS (1000 ng/L) returned to the control level after 65 days of freshwater treatment, with the female body length being significantly higher than that of the control groups, while the body length of males was significantly lower. Besides the K of the females exposed during the IDS (100 ng/L), which was significantly decreased, there were no significant differences after exposure during the IDS (as illustrated in Figure 1C).

The zebrafish that were exposed to DES in the GDS showed a significant decrease in body length at 45 dpf. However, after 45 days of freshwater maintenance, at 90 dpf, the body length and body weight of females were still significantly decreased (100 ng/L), as well as those of males exposed in the GDS (100 ng/L and 1000 ng/L). K was significantly decreased in females and males exposed during the GDS (100 ng/L).

The zebrafish that were exposed to DES in the GMS showed a significant decrease in body length at 60 dpf. However, after 30 days of freshwater maintenance, at 90 dpf, the growth parameters of all the groups returned to control levels. However, the female body length for the group exposed in the GMS (1000 ng/L) was significantly higher than that of the control groups, while K was lower for the control groups because body length increases faster than body weight.

##### 3.1.3. GSI

Although zebrafish had some time to recover after the exposure stage, the effects continued when they reached sexual maturity. Exposure led to various reductions in the GSI in females compared with that of the control groups, while the IDS and GDS exposure effects were stronger for the GMS. Exposure led to a significant decrease in the GSI during the IDS (1000 ng/L) and GDS (100 ng/L), while GMS exposure had no effect on the GSI in males. DES resulted in a more significant GSI decrease in females compared to that of males (as illustrated in Figure 2A). In general, in the first two phases, IDS and GDS, exposure to DES had stronger effects than during the GMS, and the effect on females was greater than for males.

##### 3.1.4. Sex Ratio

We observed the sex ratio of zebrafish at 90 dpf, and only the GDS (100 ng/L) exposure group was significantly higher (by 59.65%, *p* < 0.05) than the control, by about twice as many females as males (as illustrated in Figure 2B), so the results of this group were biased towards female differentiation. There was no significant difference between the zebrafish sex ratio in other exposure groups compared with that of the control group.

### 3.2. Gonad Tissues Observation

**Figure 3:**
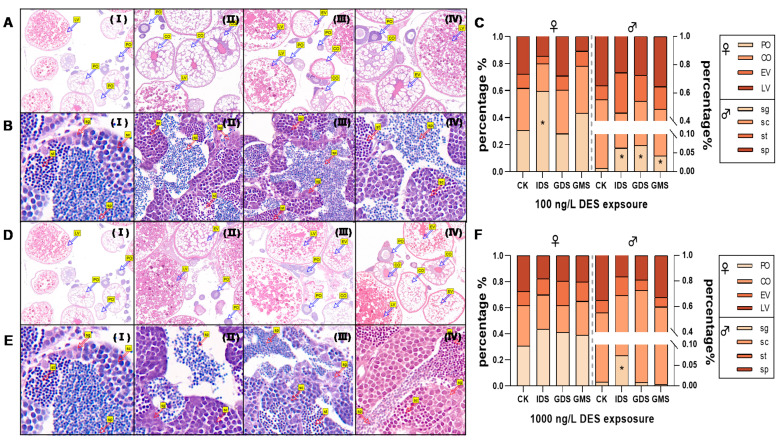
(**A**,**B**) are tissue slices from zebrafish exposed to 100 ng/L DES, (**D**,**E**) are tissue slices for 1000 ng/L DES. I–IV stand for different exposure groups (control, IDS, GDS and GMS, respectively). Percentages of egg cells and sperm cells in different development stages in females and males at 90 dpf in (**C**) (100 ng/L) and (**D**) (1000 ng/L), respectively. (**C**) and (**F**) are percentage of eggs cells and sperm cells exposed to 100 ng/L and 1000 ng/L, respectively. Values are shown as mean (*n* = 3 replicates per treatment, *n* = 2 fish per gender of each replicate with 2 cross sections). Asterisks denote significant differences between control and treatments (* *p* < 0.05, according to Dunnett’s test).LV: late vitellogenesis oocytes; EV: early vitellogenesis oocytes; CO: cortical granular oocytes; PO: perinuclear oocytes; sp: spermatogonia; sc: spermatocytes; st:spermatids; sp: sperm.

We counted the egg and sperm cells at 90 dpf for the control group and the DES exposure group exposed during the IDS, GDS, and GMS (100 ng/L (as illustrated in Figure 3A,B) and 1000 ng/L (as illustrated in Figure 3D,E)). In females, the number of PO in the early gonad development stage were approximately 59.42%, 27.93%, and 43.21%, respectively, higher than that of the control (by 30.55%), and significantly higher (*p* < 0.05) in that of the IDS 100 ng/L exposure group (as illustrated in Figure 3C). The proportion of LV at the stage of near developmental maturity decreased accordingly, and there was no significant difference due to large individual differences, to a certain extent. There was no significant difference in the other groups for 100 ng/L and 1000 ng/L DES exposure. For males, the percentage of “sg” in the IDS, GDS, and GMS groups was significantly higher (by 6.26% (*p* < 0.05), 6.96% (*p* < 0.05), and 4.17% (*p* < 0.05), respectively, in 100 ng/L) than for the control group (by 0.88%; Figure 3C). In addition, the percentage of “sg” was higher (by 7.31% in 1000ng/L (*p* < 0.05)) than in that of the control group (by 0.88%; Figure 3F).

### 3.3. Gene Expression Changes

**Figure 4:**
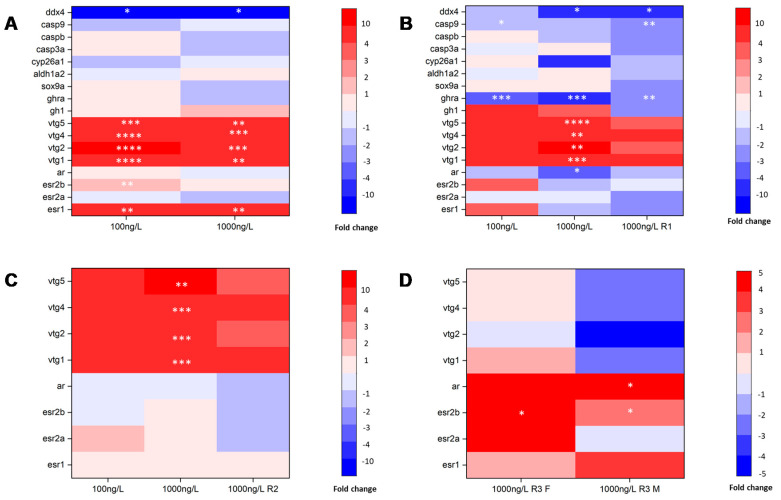
Genes for growth, HPG axis, sex hormone receptor, and gonadal differentiation and development) expression in every exposure stage. (**A**) IDS exposure group at 25 dpf; (**B**) GDS exposure group and the highest concentration (1000 ng/L) IDS exposure group at 45 dpf; (**C**) GMS exposure group and highest concentration GDS exposure group at 60 dpf; (**D**) highest concentration GMS exposure group at 90 dpf. Mean values of normalized transcript levels in each treatment were used to draw heat map (*n* = 3 replicates per treatment, *n* = 3 fish per replicate at 25 dpf, and *n* =1 fish per replicate at 45, 60, and 90 dpf). Asterisks denote significant differences between the control and treatments (* *p* < 0.05, ** *p* < 0.01, *** *p* < 0.005 and **** *p* < 0.0001, according to Dunnett’s multiple comparisons test). R1: IDS exposure group at 45 dpf. R2: GDS exposure group at 60 dpf. R3: GMS exposure group at 90 dpf. F: female. M: male.

The expressions of the genes involved in growth, the zebrafish HPG axis, sex hormone receptors, gonadal differentiation, and gonad development were determined for zebrafish exposed to DES in every phase (as illustrated in Figure 4). At 25 dpf, the IDS exposure group (100 ng/L and 1000 ng/L) displayed significantly downregulated expression of *ddx4*, which is linked to gonadal maturation and dramatically upregulated (more than 1000-folds) expression of *vtg1, vtg2, vtg4, and vtg5*, which are estrogen receptor downstream genes. Among the genes involved in the HPG axis, the genes related to estrogen signaling in the brain were significantly upregulated and the genes for sex hormone synthesis were downregulated (as illustrated in Figure 4A). At 45 dpf, the GDS exposure group (1000 ng/L) showed significant downregulation of the expression of *ar* by approximately three-fold, and *ddx4* and *ghra* by approximately four-fold, and dramatically upregulated expression of *vtg1, vtg2, vtg4, and vtg5* (only significantly for 1000 ng/L). The GDS exposure group (100 ng/L) showed significant downregulation of the expression of *casp9* by approximately two-fold, and *ghra* by approximately three-fold. After a period of recovery, the IDS exposure group (1000 ng/L) showed significant downregulation of *ddx4* by approximately five-fold, and *casp9* and *ghra* by approximately two-fold; in addition, the growth and sex hormone receptor genes were downregulated after recovery (as illustrated in Figure 4B). At 60 dpf, the expression of *vtg1, vtg2, vtg4, and vtg5* was dramatically upregulated by the exposure, while the expression of the *vtg* genes did not significantly change in the other exposure group. The sex hormone synthesis genes for the HPG axis, overall, showed a trend of downregulation (as illustrated in Figure 4C). At 90 dpf, exposure in the GMS (1000 ng/L) significantly upregulated the expression of *cyp19a1b* and *esr2b* in females and *ar* in males, and the expression of *ghra* in the pituitary was also upregulated in females and males (as illustrated in Figure 4D). In addition, we discovered that the increases or decreases in the gene expression induced by 1000 ng/L DES showed different degrees of recovery for the fish that were maintained in 0.01% acetone for a period of time after exposure. In the IDS exposure group, the significant impact on the genes *vtgs* and *ar* at 25 dpf diminished at 45 dpf. The significant gene impact at 45 dpf in the GDS exposure group mostly diminished at 60 dpf. However, the effects caused by exposure during the GMS did not seem to be alleviated at 90 dpf in the IDS and GDS groups.

## 4. Discussion

As an emerging pollutant in aquatic environments, DES may have an adverse impact on zebrafish growth and development, especially on gonad development and sex ratios. We discovered that zebrafish exposure, especially in the GDS group, significantly decreased body length, body weight, and K with increasing exposure concentrations. The significant DES inhibition effect ranking was GDS > IDS > GMS, which proved that the gonads were more sensitive during the gonadal differentiation stage. We also found that the DES inhibition effect was stronger in females than in males. In addition, the impact of DES inhibition on zebrafish gonad development may have a connection to the inhibition of growth, especially for females. Previous studies [24,27,28,29] found that the degree of gonad development and maturity may not depend on the current age, but may have a certain relationship with growth. The growth indicators include body length and weight. If body length and/or weight are affected, gonad development may be affected. In general, DES exposure may affect zebrafish gonad development by influencing body length and body weight, although body length and body weight did recover in fish kept in a freshwater environment after a period of exposure. The mating mode of male and female zebrafish comprises one-to-one mating, and changes in the sex ratio may affect the mating of males and females, resulting in a decline in the development of the population and even leading to population collapse [30]. The GDS (25–45 dpf) is a key stage for the sex differentiation of zebrafish, and DES—as a compound with estrogenic effects—could contribute to the percentage of females being higher during GDS exposure, which could decrease the development of the population [31]. In this study, DES affects the sex ratio at 100 ng/L, but not at 1000 ng/L compared with that of the control group, which may be a typical hormesis effect (overcompensation stimulation) [32]. The sex ratio in the GDS (100 ng/L) is significantly different to that of the control group, with the proportion of female and male zebrafish being 59.65 ± 9.924% and 40.35 ± 9.924% (as illustrated in Figure 2B). The degree of feminization reached approximately 70%. White, J.W et al. [31] reported that even if there was no additional EDC effect on fecundity, the sex ratio was different from 0.5, and a zebrafish population size < 60% (at best) would be unimpacted size due to feminization, resulting in population decline.

From the apparent results, DES affects the overall size of zebrafish gonads. From the tissue-level research, we found that DES may slow the maturation of egg cells in the ovary and sperm cells in the testis, and may especially inhibit testis development in IDS. The egg cells in the early stages of development are small, and the vitellogenesis oocytes are the main components of the ovary. The DES exposure group of female zebrafish ovaries had fewer LV and more oocytes in the early stages, which possibly caused the lower GSI. Compared with that of ovarian development, DES relatively inhibited testicular development, such as the number of sperm cells and their development, which may affect sustainable population development because of the sex ratio imbalance.

The GDS is a vital period for gonadal differentiation, and gonadal differentiation is influenced by many of factors. When detecting gene expressions in zebrafish, we found *vtg* series genes were highly upregulated, as there were some studies that showed that vitellogenin concentrations could be applied to the estrogen exposure endpoint, and vitellogenin concentrations and sex ratio changes could become effective biomarkers of estrone and E2 [33,34]. Sex hormones determined the sex differentiation direction. At the same time, estrogen receptors were upregulated and androgen receptors were downregulated in the zebrafish, and were biased toward differentiation in females. Dang et al. [35] reported a reversible sex ratio after depuration. The sex ratio is likely affected by the duration of exposure and depuration. We found that the GDS was easily affected by the environment, and this stage induced a stronger gene expression in DES, leading to the fish recovering more quickly in water (0.01% acetone). The GDS is an important stage to identify the potential adverse effects of DES.

## 5. Conclusions

Through our exposure experiments and depuration processes, we found obvious evidence that showed that 25–45 dpf was the most sensitive stage for zebrafish gonadal differentiation. DES exposure in zebrafish larvae, especially in the gonadal differentiation stage, caused morphological development retardation and apparently caused endocrine disruption, leading to feminization. In the future, closer attention should be paid to the impact of estrogen chemicals on the sex ratio of the zebrafish population to maintain the appropriate biological quantity and ecological balance.

## Figures and Tables

**Table 1:** Primer sequences for receptor-associated genes for RT-PCR.

Gene Name	Forward Primer (5′-3′)	Reverse Primer (5′-3′)	Gene ID
ddx4	ctcaaccacaagcatcca	agcgtccagttcttccta	ENSDARG00000014373
casp9	acgagatggacgctattc	ttgagtaggacaccaggat	ENSDARG00000004325
caspb	ggacagagacgaggagaa	agttcacagagttcagatgg	ENSDARG00000052039
casp3a	gcggatacggagactact	gattgaggcttggcatca	ENSDARG00000017905
cyp26a1	tgttctccttgccaatcg	tcacttcttctgctgttctc	ENSDARG00000033999
aldh1a2	tcttcaataacggtcaatgc	ttcctcctctgtgctctc	ENSDARG00000053493
sox9a	gacaccagcagacaacaa	gcatcagacagacacttct	ENSDARG00000003293
ghra	ccttcttcacaaccattctg	ctccaccacttctgattcc	ENSDARG00000054771
gh1,	cagttggtggtggttagtt	gcgttcctcaggcataag	ENSDARG00000038185
vtg5	agctaatgctctgcccgtta	gttcagcctcaaacagcaca	ENSDARG00000092126
vtg4	ctacaaggtggaggctctgc	ggaggacaaatcaccagcat	ENSDARG00000078429
vtg2	tactttgggcactgatgcaa	agacttcgtgaagcccaaga	ENSDARG00000055809
vtg1	ctgcgtgaagttgtcatgct	gaccagcattgcccataact	ENSDARG00000092233
ar	gtctattaagagccgcctat	caccgcaacaagttcatc	ENSDARG00000067976
esr2b	ttgtgttctccagcatgagc	ccacatatggggaaggaatg	ENSDARG00000034181
esr2a	agcattagccttgttagca	atgttgtcacggatgtcat	ENSDARG00000016454
esr1	ggtccagtgtggtgtcctct	cacacgaccagactccgtaa	ENSDARG00000004111
